# Crystallographic information of intermediate phases in binary Mg–*X* (*X*=Sn, Y, Sc, Ag) alloys

**DOI:** 10.1016/j.dib.2015.05.011

**Published:** 2015-05-27

**Authors:** Dongyan Liu, Xuefeng Dai, Xiaohong Wen, Gaowu Qin, Xiangying Meng

**Affiliations:** a College of Sciences, Northeastern University, Shenyang 110819, China; b Key Laboratory for Anisotropy and Texture of Materials (MOE), Northeastern University, Shenyang 110819, China

## Abstract

The compositions and structures of thermodynamically stable or metastable precipitations in binary Mg-*X* (*X*=Sn, Y, Sc, Ag) alloys are predicted using ab-initio evolutionary algorithm. The geometry optimizations of the predicted intermetallic compounds are carried out in the framework of density functional theory (DFT) [1]. A complete list of the optimized crystallographic information (in cif format) of the predicted intermetallic phases is presented here. The data is related to “Predictions on the compositions, structures, and mechanical properties of intermediate phases in binary Mg–*X* (*X*=Sn, Y, Sc, Ag ) alloys” by Liu et al. [2].

Specifications Table

**Table:** 

Subject area	Physics, Chemistry, Material
More specific subject area	Computational condensed matter, Computational materials science
Type of data	Text, Crystallographic information in cif format
How data was acquired	By *ab*-initio evolutionary algorithm Universal Structure Predictor: Evolutionary Xtallography (USPEX) [3–6] and *ab*-initio total energy program Vienna Ab initio simulation package (VASP) [7–9]
Data format	Raw
Experimental factors	–
Experimental features	First-Principles simulations
Data source location	Northeastern University, Shenyang, China
Data accessibility	Data is available with this article and is related to Liu et al [2].

Value of the data

**Table:** 

• We provide comprehensive crystallographic information for binary Mg-*X* (*X*=Sn, Y, Sc, Ag) alloys system.
• The data is expected to help in novel material designing and exploring.
• We propose a new idea in theoretical material designing.

## 1 Computational methods

### 1.1 Structures prediction

Combining the evolutionary algorithm USPEX with the *ab*-initio total energy program VASP, we predicted thermodynamically stable or metastable structures in binary Mg-*X* (*X*=Sn, Y, Sc, Ag) alloy systems. The first-principles pseudopotential Perdew–Burke–Ernzerhof (PBE) approach [10] was chosen as the exchange and correlation functional. To construct the pseudopotentials (PPs) of Mg, Sn, Y, Sc, and Ag elements, we treat their respective 3*s*^2^, 5*s*^2^5*p*^2^, 4*s*^2^4*p*^6^4*d*^1^5*s*^2^, 3*s*^2^3*p*^6^3*d*^1^4*s*^2^, and 4*d*^10^5*s*^1^ orbitals as valence electronic configurations throughout this work. The projector augmented wave (PAW) [11,12] method was adopted, and the plane-wave kinetic energy cutoff was set to 600 eV. The Brillouin zone was sampled with a resolution of 2*π*×0.05, with that all runs found the minimum-enthalpy structures much earlier. In the present work, the maximum total numbers of atoms in the unit cell were limited to 16. The first generation of possible structures was created randomly, then a new generation was created through heredity, lattice mutation and permutation of atoms. The most favorable structures were transferred into the next generation. The total number of generations was set to 50, and USPEX searching automatically terminated when predicted phases keep unchanged for 20 generations.

We define stables as those compositions having lower free energy than any isochemical mixture of other compounds or pure elements, while metastables as compositions whose formation enthalpies are close to but ~0.1 eV/atom higher than the convex hull linked by the stables. We expect that the metastables can gain ~0.1 eV/atom energy at the melting temperatures of most magnesium alloys (~1000 K), and the extra heat and vibration energies may result in a reversed free energy order and make matestables to be stables at elevated temperatures. Such a energy standard for metastables (0.1 eV/atom) was also adopted in the high-throughput (HT) calculations [13].

### 1.2 Geometry optimization

The geometry optimizations of the predicted binary Mg-*X* (*X*=Sn, Y, Sc, Ag) compounds were carried out in the framework of density functional theory (DFT) [1]. In this step, the PBE method was employed as the exchange-correlation functional, and an expansion of the electronic wave functions in plane waves with a kinetic-energy cutoff of 600 eV was adopted. Brillouin-zone integrations were performed using 8×8×8 Monkhorst-Pack K-point meshes [14]. The convergence criteria for energy and Hellmann-Feynman force was set to 10^−8^ eV and 1 meV/Å, respectively.

## Supporting information

Complete list of the optimized crystallographic information (in.cif format) of the predicted intermetallic phases for Mg-*X* (*X*=Sn, Y, Sc, Ag).
